# Changes in Breast Cancer Cell Electrophysiology in Response to Culture Across a Wide Range of pH: Dielectrophoresis and ζ-Potential

**DOI:** 10.3390/mi17080902

**Published:** 2026-07-28

**Authors:** Mary Krystelle Catacutan, Sungmun Lee, Michael Pycraft Hughes

**Affiliations:** 1 Department of Biomedical Engineering and Biotechnology, Khalifa University of Science and Technology, Abu Dhabi 127788, United Arab Emirates; marykrystelle.academia@gmail.com (M.K.C.); sung.lee@ku.ac.ae (S.L.); 2 Healthcare Engineering Innovation Group, Department of Biomedical Engineering and Biotechnology, Khalifa University of Science and Technology, Abu Dhabi 127788, United Arab Emirates; 3 Biotechnology Center, Department of Biomedical Engineering and Biotechnology, Khalifa University of Science and Technology, Abu Dhabi 127788, United Arab Emirates

**Keywords:** breast cancer, pH, membrane potential, zeta potential, dielectrophoresis

## Abstract

To survive, cells are able to adapt to a wide range of adverse conditions, such as varying pH from optimal (~7.4). They do this through mechanisms including acid-sensing ion channels, which alter cytosolic ion content and thus the cell’s electrophysiological profile. However, the impact of this adaptation on cellular electrophysiology remains unexplored. We investigated the effects of culture at a range of extracellular pH on the electrophysiological features of breast cancer cell lines MDA-MB-231 and MCF-7. Cells were subject to an acid–neutral–base pH from 3.0 to 9.2, after which their membrane potential (*V_m_*), cytoplasm conductivity *σ_cyto_*, effective membrane conductance *G_eff_*, and ζ-potential were measured. Cells were also analyzed after permeabilization, to examine whether observed changes were due to cell surface chemistry, or to *V_m_*. Both cell lines exhibited different electrophysiological phenotypes in acidic environments (pH < 6.7); MDA-MB-231 exhibited statistically significant differences in ζ-potential, *V_m_*, *G_eff_* and *σ_cyto_*; MCF-7 only exhibited significant differences in *σ_cyto_*. These findings suggest cells adapt to acidic microenvironments by altering *V_m_* and potentially ζ-potential, reducing the extracellular potential, and hence potentially lowering proton concentration at the extracellular membrane surface. This offers new insights into potential therapeutic avenues to target the pH-dependent adaptations of cancer cells.

## 1. Introduction

When dividing and multiplying, cells must sometimes endure a suboptimal environment. Whilst cells are optimized to grow in ideal conditions of nutrient and gas availability, ionic strength, and temperature, they are also able to grow when some of these conditions are not ideal; “life finds a way”. For example, whilst the optimal pH for cell growth is 7.4, cells in the tumor microenvironment thrive in extracellular pH (pHe) of around pH 6.7 [1,2,3] caused by altered cellular metabolism [4,5,6,7], which actively contributes to oncogenic processes such as metastasis and angiogenesis [5]. Cells adapt to extracellular pH in many ways; as a first line of defense, they can expel excess protons, or increase intracellular proton consumption, whilst as a last resort, cells undergo metaplasia, adapting the phenotype to the surroundings [8]. Altered pHe is also known to modulate specific ion channel activities. Ion channels regulate the intracellular concentration of specific ion species in response to a range of intrinsic and extrinsic cues. This alters the passage of ions down a concentration gradient, but also alters the cell’s membrane potential, *V_m_*.

It has been known for some time that *V_m_* is intimately connected to the cell’s extracellular surface potential [9,10,11,12]. This has recently been shown to be due to *V_m_* mechanistically altering the surface charge, rather than the other way around, such that dynamic alteration of *V_m_* produces simultaneous alterations in the surface potential [13,14]. This has been shown to alter the concentration of both ions [13] and protein interactions [15] at the cell surface, in line with the Poisson–Boltzmann equation [16]. If the surface potential becomes more negative, for example, it increases the concentration of cations at the surface and diminishes anions. Since the proton is a positively charged hydrogen ion, we speculated that the observed ion channel activity at suboptimal pH may represent an additional defense mechanism in the face of suboptimal pHe during cell culture. By altering its intracellular ionic environment, the cell could alter both *V_m_* and ζ-potential, which may then alter the extracellular acid concentration. To test this hypothesis, we cultured two breast cancer cell lines to confluence in conditions with a broad range of pH, from 3.0 to 9.2, and then analyzed them to examine their electrophysiology.

Cell electrophysiology is most commonly measured using patch clamp, a technique whereby a saline-filled glass micropipette is maneuvered to the surface of the cell being analyzed, using a micromanipulator [17]. Once in contact, the cell is drawn to the pipette opening via a small negative pressure; when a seal is formed, a puncture in the membrane can be created by dropping the pressure further. This allows for a “whole cell” measurement of the cell, allowing its membrane capacitance and conductance to be measured in addition to *V_m_* [18]. This is a difficult and slow process; patch clamp requires skilled operators, and patching a single cell can take more than an hour. This leads to reduced data throughput, as well as operator bias, where it is often the larger cells, rather than the median-sized cells, which are patched.

To overcome this, a new method has been developed, using dielectrophoresis (DEP) [19]. DEP is a phenomenon of induced motion in non-uniform electric fields across a range of frequencies, which yields the membrane and cytoplasm conductivity and membrane capacitance at the conductivity of the medium in which the cell is suspended [20], whilst analyzing at multiple conductivities further allows the estimation of membrane potential [21]. This allows the rapid analysis of large numbers of cells; a typical measurement requires approximately one minute, whilst determining the mean properties of ca. 20,000 cells [22].

Similarly, it is possible to measure a correlate of the surface potential. The electrical properties of the surface itself are hidden behind the ions and water molecules that are hydrodynamically bound to it, but their influence can be measured at the interface between this stagnant layer and the freely moving bulk medium. The voltage at this hydrodynamic plane of shear (approximately 1–2 nm from the cell surface) is called the ζ-potential [23]. The ζ-potential is commonly measured in materials science, where it describes whether particles in solution remain dispersed, or flocculate. A standard method of measuring it is electrophoretic light scattering (ELS), where the interaction between the cell and a uniform, invariant electric field causes the cell to move, which is then measured using Doppler analysis of an incident laser [24]. In this paper, we use DEP and ELS to study the effect on cellular electrophysiology of culture at a range of pH. This study shows that cells do alter their electrophysiology to adapt to a range of pHe, with alterations in passive electrical properties, *V_m_*, and ζ-potential.

## 2. Materials and Methods

### 2.1. Cell Culture Procedure

Two breast cancer cell lines, MDA-MB-231 (Cat. No. C0006002) and MCF-7 (Cat. No. C0006008), were obtained from AddexBio (San Diego, CA, USA) to test the effects of pH on the electrophysiological properties of cancer cells. MDA-MB-231 is a triple-negative subtype, making it highly aggressive and metastatic [25]. MCF-7 is a luminal A breast cancer, which is generally less aggressive with lower metastatic potential [26]. The MDA-MB-231 cells were cultured in DMEM (Elabscience, Houston, TX, USA) and the MCF-7 cells were cultured in DMEM/F-12 (Elabscience), both supplemented with 10% fetal bovine serum (FBS) and 1% penicillin–streptomycin at 37 °C and 5% CO_2_. MDA-MB-231 (300,000 cells/mL) and MCF-7 (300,000 cells/mL) were seeded in 6-well plates. Upon reaching approximately 90% confluence, the cells were incubated in the pH-modified medium for 24 h. Following treatment, the cells were trypsinized, neutralized with culture medium, and centrifuged. The cell pellet was resuspended in 8.5% (*w*/*v*) sucrose and 0.5% (*w*/*v*) dextrose for further analysis. Cell size and viability were determined using an automated cell counter, Countess 3 FL Automated Cell Counter (Thermo Fisher Scientific, Waltham, MA, USA), which was also used to measure cell radii for use in DEP calculations. Cells were grown directly in 6-well plates and visualized using the Carl Zeiss Axio Vert. A1 inverted microscope (ZEISS, Oberkochen, Germany) for morphological assessment.

### 2.2. Modifying pH Medium

To create a range of pH conditions for treatment, the culture medium was adjusted to specific pH levels by adding 1 N hydrochloric acid (HCl) or 1 N sodium hydroxide (NaOH). The pH was monitored using a pH meter (Orion Star A211, Thermo Fisher Scientific) and adjusted to the desired values (pH 3.0, 4.0, 5.0, 6.7, 7.4, 8.4, and 9.2). The adjusted medium was thoroughly mixed to ensure uniform pH distribution before being added to the cells. The physiological and tumor microenvironment pH range (6.7–7.4) served as the homeostatic baseline and reflects the mildly acidic conditions typical of the solid tumor microenvironment. An extremely acidic pH (3.0–5.0) was used to drive the cell membrane toward its isoelectric point, as extreme protonation induces maximal gating of acid-sensing ion channels. Alkaline pH (8.4–9.2) was utilized to ensure complete deprotonation of surface functional groups to establish the upper boundary of the cell membrane’s electronegativity.

### 2.3. Permeabilization

In order to measure cells in the absence of a membrane potential, cells were also measured following permeabilization. Immediately before measurement, the cells were incubated with 2 μg/mL Tween-20, a mild non-ionic detergent, for 5 min at room temperature. The efficacy of the permeabilization protocol was measured by incubating with a 0.4% trypan blue solution. Microscopic inspection indicated that the majority of the cells absorbed the dye, signifying a loss of membrane integrity and disruption of the membrane potential.

### 2.4. Isosmotic Medium

An isosmotic solution was prepared by dissolving 8.5% (*w*/*v*) sucrose and 0.5% (*w*/*v*) dextrose in deionized water. To this, phosphate-buffered saline (PBS) was added by pipette whilst the medium was stirred and measured using a conductivity meter (Orion Star A214 (Thermo Fisher Scientific) calibrated with standard reference solutions) until the desired conductivity was achieved. Media were prepared at two specific conductivity levels, 43 mS/m (referred to here as “low conductivity”) and 143 mS/m (hereafter “high conductivity”).

### 2.5. Measurement 

Replicates. All measurements were performed on three biological replicates. For each biological replicate, all measurements were taken three times.

Morphology. The morphological changes in the MDA-MB-231 and MCF-7 cells in response to varying pH values were assessed using the Carl Zeiss Axio Vert. A1 inverted microscope after a 24 h incubation period.

ζ-potential. The ζ-potential of the cells was measured using a Malvern Zetasizer (Malvern Instruments, Worcestershire, UK). Prior to measurement, the sample was resuspended in an isosmotic solution and was placed in a folded capillary cell. The measurement was performed at a temperature of 25 °C.

DEP. Measurements of cytoplasm conductivity *σ_cyto_* and effective membrane conductance per unit area *G_eff_* were determined using a DEPtech 3DEP (Heathfield, UK) [22]. Before measurement, the samples were resuspended in an isosmotic solution at a concentration of approximately 10^6^ per ml, determined using an Invitrogen Countess 3 automated cell counter (Thermo Fisher, Waltham, MA, USA). Approximately 85 μL of cell solution was aspirated using a blunt-needle syringe. The samples were injected into DEP Chips 806 and were aligned with the electrodes on the 3DEP machine. Mean *σ_cyto_* was determined using a best-fit Clausius–Mossotti model, which has been demonstrated to produce results comparable to gold-standard electrophysiology methods such as patch clamp.

Membrane potential. The *V_m_* of the cancer cells was determined using the DEP-based approach [21] for determination of the absolute value of resting membrane potential, using the change in DEP-derived cytoplasm conductivity (*δσ_cyto_*) for the equivalent change in medium conductivity (*δσ_med_*). This relationship is expressed as:

(1) $$V_{m}=-\left(\frac{RT}{F}\right)\left|ln\left(\frac{\delta \sigma_{cyto}}{\delta \sigma_{med}}\right)\right|-\Psi_{offset}$$

where *R* represents the ideal gas constant, *T* is the absolute temperature, *F* is the Faraday constant, and *Ψ*_offset_ is −12 mV. The technique has been demonstrated to yield estimated values of RMP consistent with patch clamp across a wide range of cell types, including monocytes, platelets, red blood cells, Jurkat and HeLa cancer lines, mesenchymal stem cells [27], Candida Albicans strains [28] and primary chondrocytes, as they transform to fibroblasts [29].

### 2.6. Statistical Analysis

Statistical significance between independent pH groups (6.7 < pH ≥ 6.7) was compared using Welch’s two-sample *t*-tests applied separately within each subgroup defined by the combination of cell line, cell state, and *σ_med_* level. Significance was defined as *p* < 0.05 (*), *p* < 0.01 (**), *p* < 0.001 (***), and *p* < 0.0001 (****). Normality of the data distribution within each experimental condition was evaluated using the Shapiro–Wilk test; because the data did not meet the assumption of normality, a non-parametric framework was used for further analysis.

The effect of pH on each biophysical parameter (membrane potential, cytoplasm conductivity, membrane conductance, and ζ-potential) was evaluated without assuming linear response, using non-parametric Spearman’s rank-order correlation within each subgroup defined by cell line (MCF-7, MDA-MB-231), cell state (intact, permeabilized), and condition (low- and high-conductivity medium). Spearman’s rank correlation coefficient is a robust non-parametric measure for monotonic dependency. This trend is visually represented in the figures by significance asterisks (*), denoting the statistical strength of the monotonic association between pH and the electrical parameter. Pairwise comparisons between pH levels were performed using Dunn’s test, following a non-parametric approach. The Benjamini–Hochberg (BH) procedure was used to adjust the *p*-values (p_adj_) to account for the risk of Type I errors inherent to multiple independent comparisons and controls for False Discovery Rate (FDR). All results are reported as adjusted *p*-values, and only significant trends are shown in the figures.

To assess the relationship between *V_m_* and ζ-potential, mean values of the pH subgroups were aggregated, which quantifies the degree of biophysical coupling independent of the pH stimulus. The strength of the relationship is presented using the Spearman rank correlation coefficient (ρ), to identify the non-linear monotonic associations between these parameters. The resulting association was visualized using locally estimated scatterplot smoothing (LOESS) regression to capture the non-linear trajectories of these parameters.

### 2.7. Gene Expression for Acid-Sensing Ion Channels (ASICs)

A curated list of protein-coding ion channel genes was obtained from the HUGO Gene Nomenclature Committee (HGNC) database. The ion channel gene expression data for the cancer cell lines were retrieved from the OmicsExpressionProteinCodingGenes dataset (DepMap, Broad (2025), DepMap Public 25Q2), which provides standardized RNA-seq expression values from the Cancer Cell Line Encyclopedia (CCLE). The expression matrix for the cancer cells was extracted, and the gene names were annotated with their respective ion channel types. For each gene, expression values were averaged across replicates within each cell line. To quantify differential expression, the fold-change values were log2-transformed to facilitate interpretation, and genes with zero or underdefined variance were excluded. Data were visualized as a horizontal bar plot, with positive values indicating higher expression in MDA-MB-231 and negative values indicating higher expression in MCF-7. All data processing, statistical analyses, and visualization were performed using the R programming language (version 4.3.1) within the RStudio integrated development environment (IDE).

## 3. Results

### 3.1. Effect of pH on Cell Morphology

**Figure 1:**
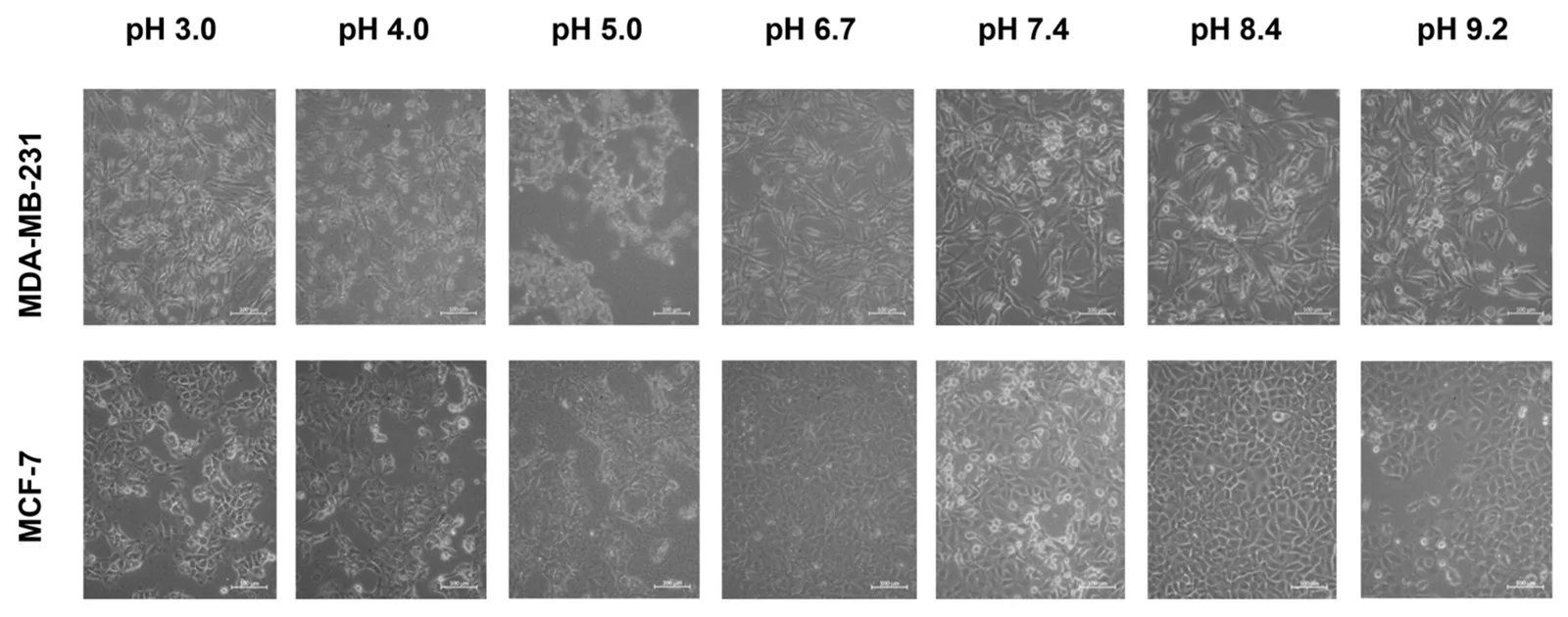
The effect of extracellular pH on the morphology of MDA-MB-231 and MCF-7 cell lines (scale bar = 100 μm, applies to all panels). Each cell line was incubated at pH levels ranging from 3.0 to 9.2, highlighting pH-dependent changes in cellular structure. MDA-MB-231 showed minimal morphological alteration across the different pH levels. However, MCF-7 exhibited morphological changes, which are most apparent at pH 5.0.

Figure 1 shows representative images of cells following incubation at different pHe. Both MDA-MB-231 and MCF-7 exhibited morphological resilience across all tested pH environments. MDA-MB-231 maintained its elongated appearance and remained adherent, and MCF-7 retained its epithelial-like appearance. The largest alterations occurred in both cell lines at and below pH 5.0, where some cell detachment was observed in both lines, and MCF-7 showed occasional signs of rounding and reduced spreading. At alkaline conditions, both cell types maintained adhesion and exhibited no morphological change compared to neutral pH.

### 3.2. Effect of pH on Cytoplasm Conductivity and Membrane Conductance

**Figure 2:**
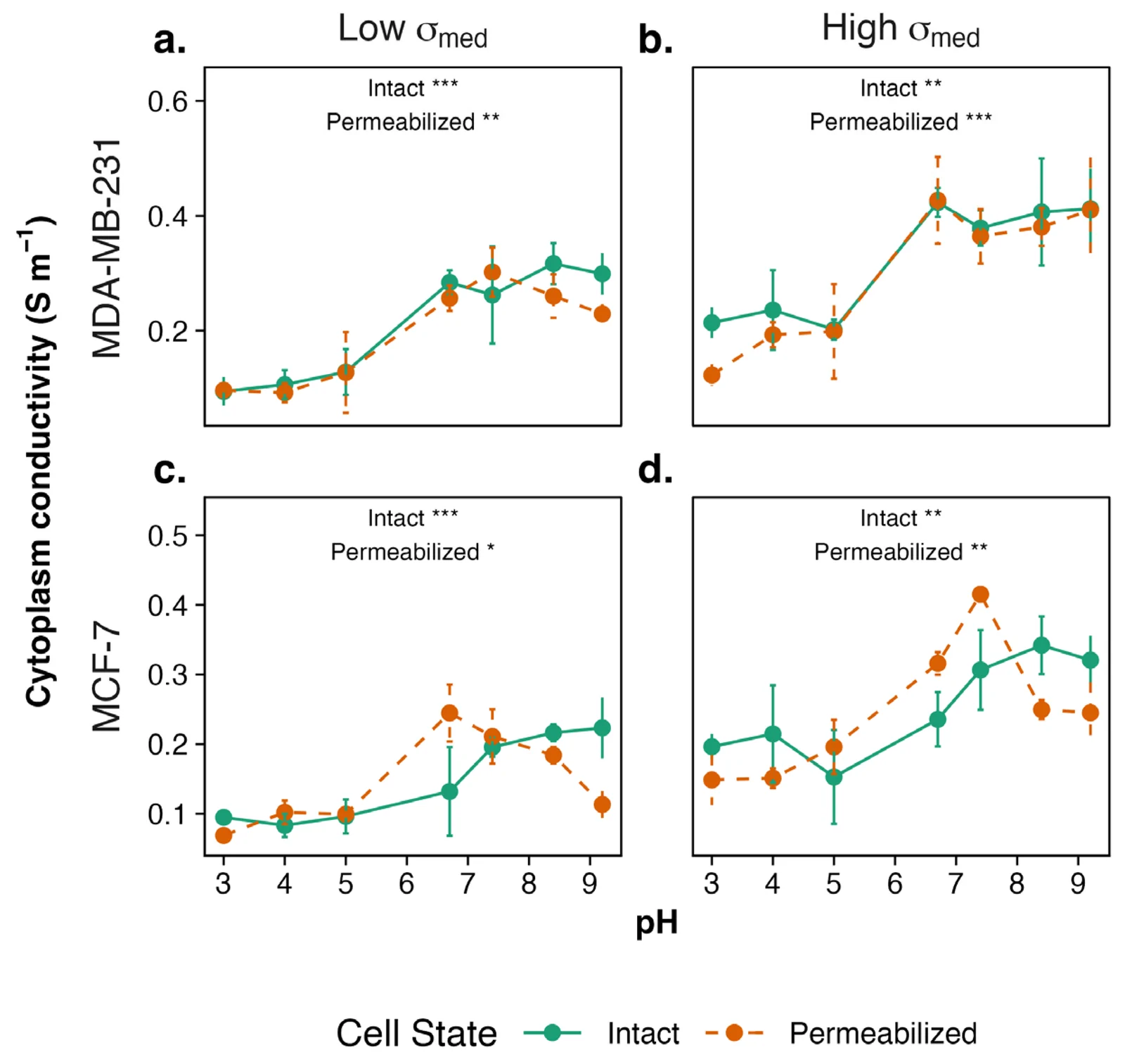
The cytoplasmic conductivity *σ_cyto_* of intact and permeabilized MDA-MB-231 cells in (**a**) low- and (**b**) high-conductivity medium and of intact and permeabilized MCF-7 cells in (**c**) low- and (**d**) high-conductivity medium measured for cells cultured across pH 3.0–9.2. Both intact and permeabilized cancer cells show a gradual increase in conductivity with increasing pH. While the pH-dependent trend is preserved in both groups, overall cytoplasmic conductivity values are elevated. Data are presented as mean ± SEM (*n* = 3 biological repeats × 3 technical repeats). Statistical significance was assessed within each group across pH values (* *p* < 0.05, ** *p* < 0.01, *** *p* < 0.001).

To determine the effect of pHe on cytoplasm conductivity *σ_cyto_*, measurements were performed on cells cultured across a pH range of 3.0 to 9.2 for both intact and permeabilized cells. Spearman rank-order correlation revealed a significant monotonic association between pH and *σ_cyto_* in intact and permeabilized cancer cells. Considering MDA-MB-231 first (Figure 2a,b), both intact and permeabilized cells showed a pH-dependent trend in *σ_cyto_*, which was relatively low at acidic pH (3.0–5.0), increased around neutral pH to a higher value, and maintained that value at alkaline pH (7.4–9.2). Both intact and permeabilized cells showed a gradual increase from pH 3.0 to pH 6.7, followed by a gradual decrease from pH 8.4 to pH 9.2. In high-conductivity medium, MDA-MB-231 also followed a similar trend; however, a gradual decrease in *σ_cyto_* was seen as pH increased past pH 8.4 in intact cells. Overall, δ*σ_cyto_* increased in higher medium conductivity at all pH levels.

For intact MCF-7 (Figure 2c,d), *σ_cyto_* remained stable between pH 3.0–5.0 followed by a slight increase at pH 6.7, with conductivity continuing to rise through pH 9.2. Permeabilized cells, in contrast, followed a similar trend as MDA-MB-231, with *σ_cyto_* remaining stable at low pH and significantly increasing at pH 6.7 and pH 7.4 (*p* < 0.05) before showing a gradual decrease through pH 9.2. Higher medium conductivity resulted in only minor shifts in *σ_cyto_* at each pH value. The overall pH-dependent trend was observed across all medium conditions, with intact MCF-7 cells experiencing a slight decrease in *σ_cyto_* at pH 5.0 and permeabilized cells exhibiting a gradual significant increase between pH 3 and pH 7.4.

**Figure 3:**
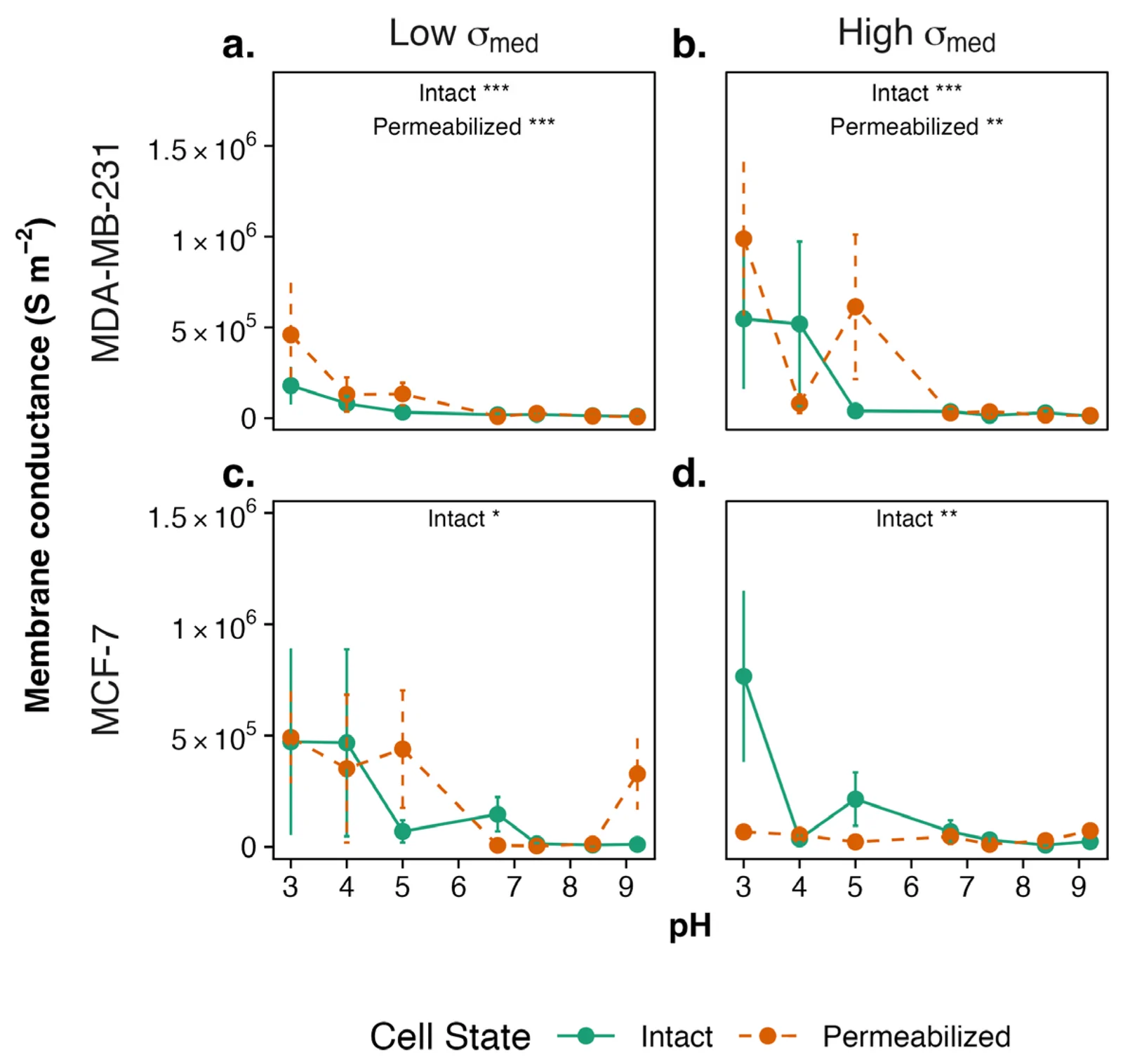
The membrane conductance *G_eff_* of intact and permeabilized MDA-MB-231 cells in (**a**) low- and (**b**) high-conductivity medium and of intact and permeabilized MCF-7 cells in (**c**) low- and (**d**) high-conductivity medium measured across pH 3.0–9.2. Conductance remains near zero at neutral and alkaline pH with a slight increase and a higher variability observed at acidic pH. Data are presented as mean ± SEM (*n* = 3 biological repeats × 3 technical repeats). Statistical significance was assessed within each group across pH values (* *p* < 0.05, ** *p* < 0.01, *** *p* < 0.001).

Membrane conductance of intact and permeabilized MDA-MB-231 and MCF-7 was measured for cells cultured across the pH range, and cells under low- and high-conductivity medium conditions (Figure 3). Cells cultured in alkaline-to-neutral pH exhibited low, near-zero values of *G_eff_*. Conversely, at acidic pH, conductance values exhibited high levels of variability; consequently, the data showed large standard errors of the mean and no statistically significant differences between conditions. A statistically significant change is observed at high conductivity for intact MDA-MB-231 at low pH (3.0 and 4.0) and high pH (7.4 and 9.2), indicating that extreme acidic and ionic stress conditions produce measurable changes in membrane conductance. Conversely, intact MCF-7 in high conductivity medium at pH 3.0 is significantly different than at pH 8.4. Overall, while intact MCF-7 cells exhibit a statistically significant monotonic association between pH and *G_eff_*, permeabilized cells do not show a consistent monotonic trend, suggesting that the MCF-7 *G_eff_* response to a pH shift requires an intact membrane.

### 3.3. Effect of pH on ζ-Potential

Surface chemistry is sensitive to pH owing to the protonation and deprotonation of surface functional groups such as hydroxyl and carboxylate groups [30]. At low pH, these groups are normally protonated, resulting in a net positive surface charge; when pH rises, deprotonation occurs, resulting in an increasingly negative charge [31]. This transition occurs through the isoelectric point (IEP), which is the pH where the net surface charge is zero [32]. Surfaces around the IEP exhibit less electrostatic repulsion, frequently leading in greater particle aggregation since van der Waals attraction dominates interparticle interactions [33]. The IEP is an important parameter in colloid and interface research as it determines dispersion stability [34] and the adsorption process, and the exploitation of the isoelectric point in the analytical technique of “isoelectric focusing” is widely used for measuring the electrical charge of macromolecules.

**Figure 4:**
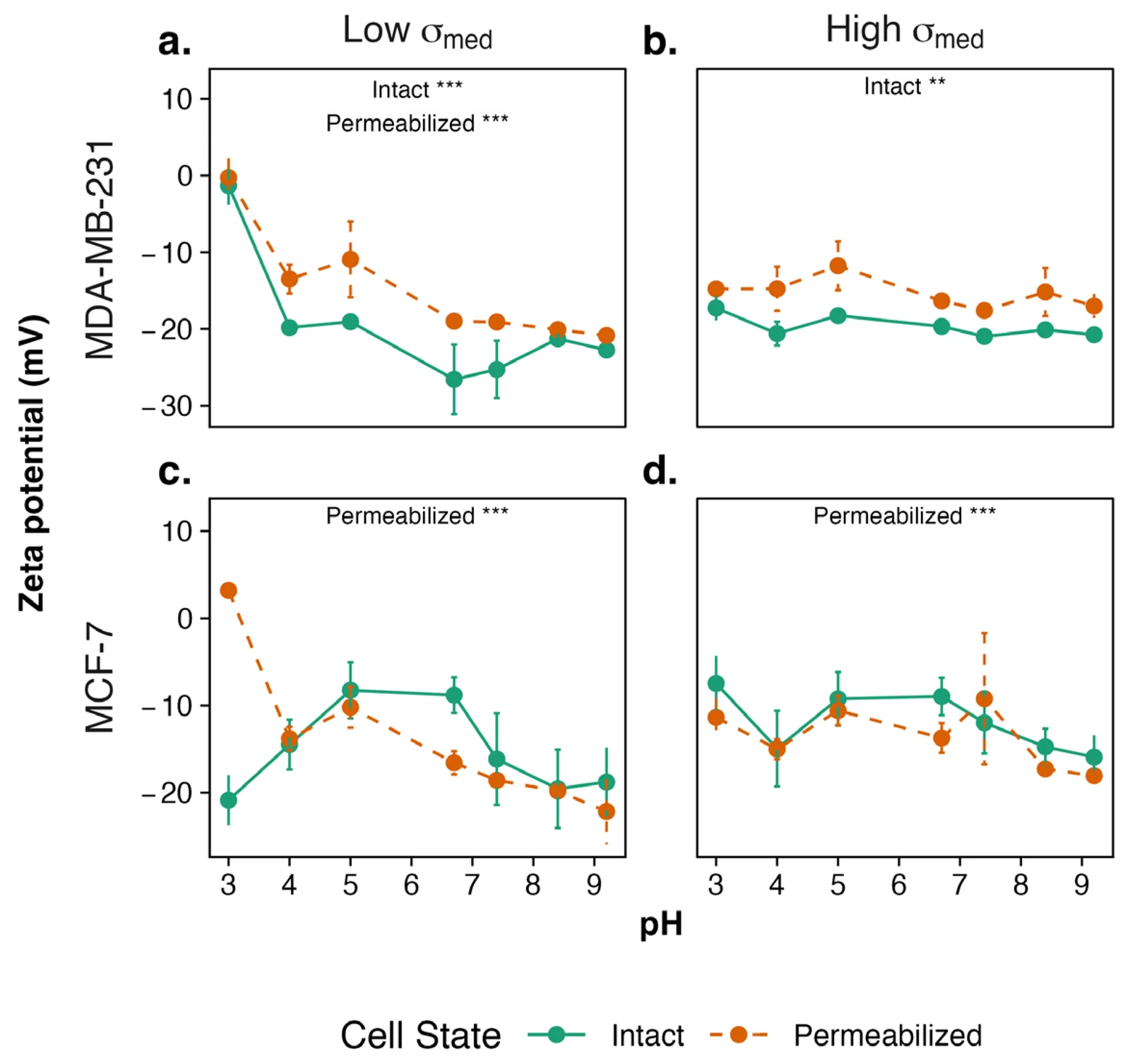
The ζ-potential of intact and permeabilized cells across pH. (**a**) In low-conductivity medium, both intact and permeabilized MDA-MB-231 exhibits a significant monotonic association in ζ-potential with increasing pH. (**b**) In high-conductivity medium, ζ-potential remains stable across pH for intact and permeabilized cells, with no significant differences observed. (**c**) In low- and (**d**) high-conductivity medium, intact MCF-7 did not follow a monotonic association. Data are presented as mean ± SEM (*n* = 3 biological repeats × 3 technical repeats). Statistical significance was assessed within each group across pH values (** *p* < 0.01, *** *p* < 0.001).

Intriguingly, the ζ-potential of intact and permeabilized cells was influenced significantly after culture at different pH values in low-conductivity medium (*p* < 0.05), despite the measurements being taken at neutral pH (Figure 4). In low-conductivity medium, intact MDA-MB-231 showed ζ-potential became significantly more negative with increasing pH, beginning at around zero for cells cultured in pH 3.0 and reaching a peak for those cultured at pH 6.7. Permeabilized MDA-MB-231 exhibited a similar pH dependence but maintained a consistently less negative ζ-potential across the range. This suggests that under reduced ionic strength, the electrostatic properties of the cell surface are more sensitive to pH-dependent changes. In high-conductivity medium, the profile was similar across the pH range with the exception of the depolarization observed at the lowest pH; the ζ-potential values for both intact MDA-MB-231 cells became significantly more negative, suggesting enhanced ion mobility or altered ionic interactions at the cell surface. However, no significant differences were observed in permeabilized cells across pH, indicating stable surface characteristics in ionic environments.

In a low-conductivity medium, the intact MCF-7 (Figure 4b) ζ-potential did not display a monotonic relationship (*p* > 0.05) with pH for intact cells. ζ-potential was more negative at low pH, less negative near neutral, and more negative again under alkaline conditions. Meanwhile, permeabilized MCF-7 showed a monotonic relationship, with statistical significance between pH 3 and pH 7.4, 8.4 and 9.2 (*p* < 0.05), indicating a consistent change in surface charge with pH. In high conductivity medium, ζ-potential followed a U-pattern for both intact and permeabilized MCF-7, with significant monotonic association observed only in permeabilized cells.

### 3.4. Effect of pH on Membrane Potential (V_m_)

**Figure 5:**
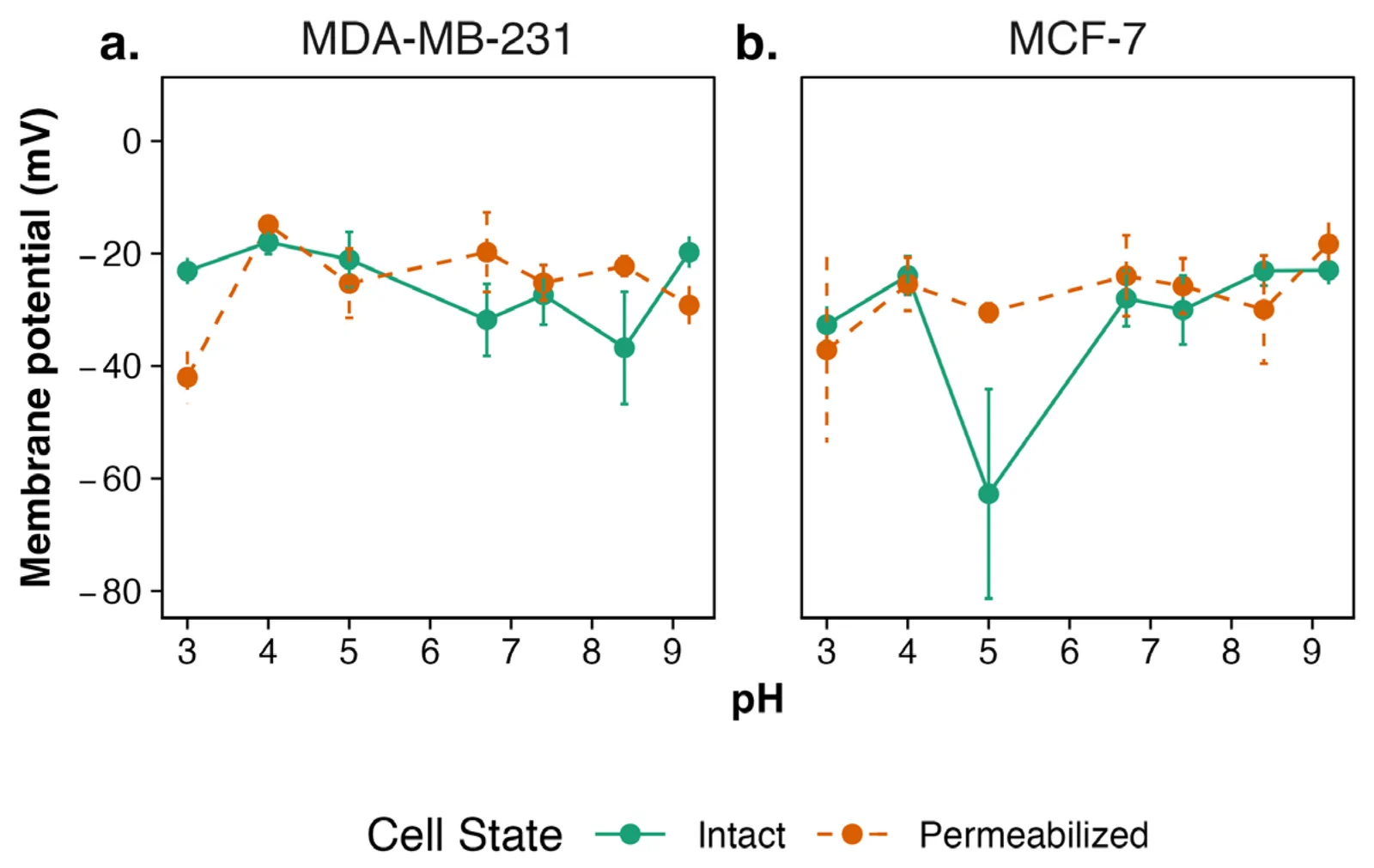
Membrane potential across pHe in (**a**) MDA-MB-231 and (**b**) MCF-7 cell lines. Membrane potential was derived from DEP-based cytoplasmic conductivity measurement using the method by Hughes et al. [21]. Data are presented as mean ± SEM (*n* = 3 biological repeats × 3 technical repeats).

Membrane potential (*V_m_*) was estimated across the range of pHe values using Equation (1). Intact and permeabilized MDA-MB-231 (Figure 5a) maintained membrane polarization across the pH range, with a hyperpolarization near neutral pH, likely reflecting active ion regulation. In MCF-7 cells (Figure 5b), *V_m_* measurements showed a consistent trend in both intact and permeabilized cells, with values remaining relatively stable except at pH 5.0, where a clear hyperpolarization occurred. However, this shift was not statistically significant in both intact and permeabilized MCF-7 cells. This indicates that the underlying physicochemical shift is not amplified in the absence of intact membranes.

**Figure 6:**
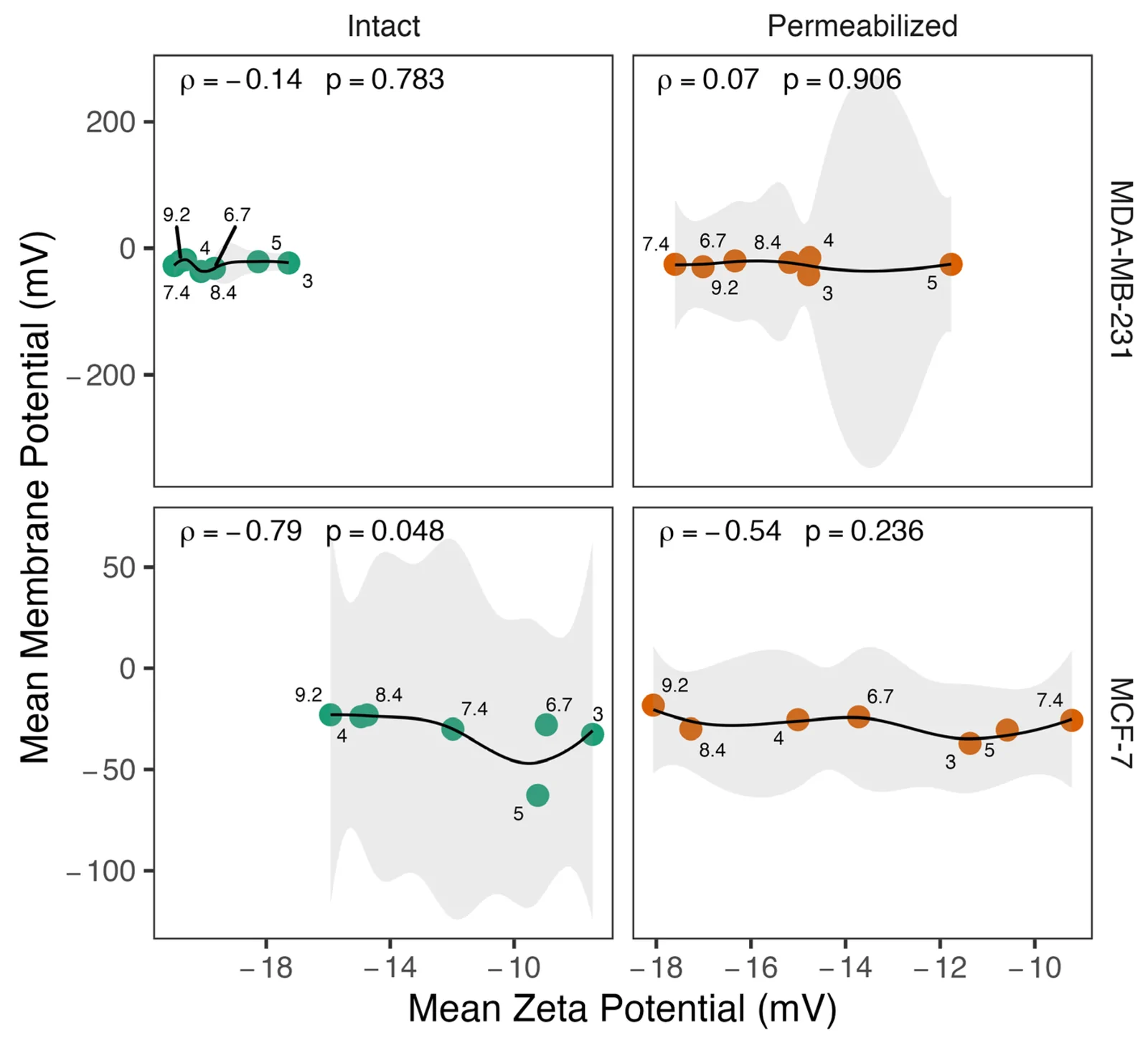
The relationship between mean membrane potential and mean ζ-potential across varying pH environments. The scatter plot shows the trajectory of intact (green) and permeabilized (orange) MCF-7 and MDA-MB-231 cells. Data points represent the mean values for specific pH conditions (labeled), and Spearman’s rank correlation coefficient (ρ) indicates the strength of the monotonic relationship.

**Figure 7:**
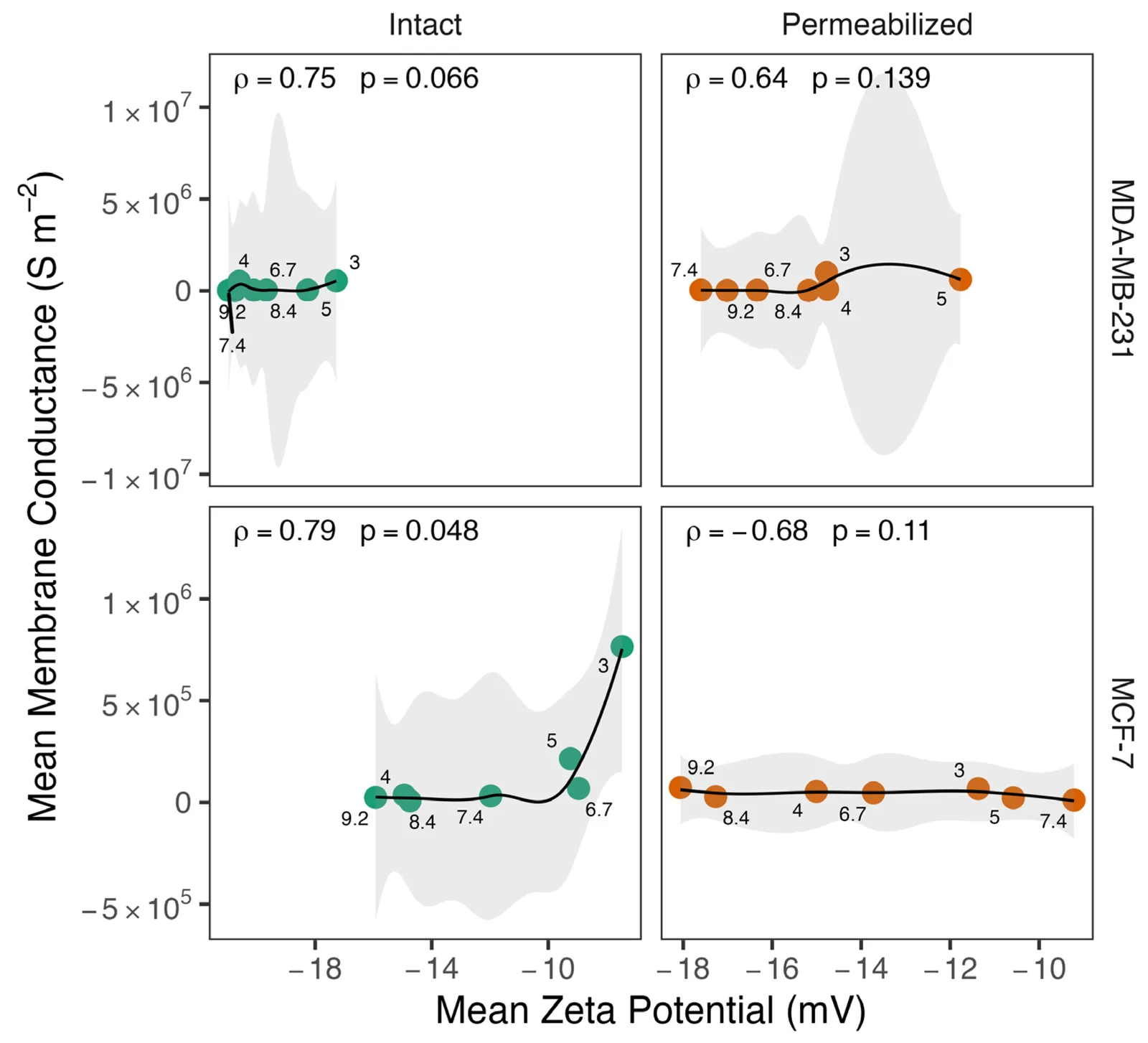
Relationship between mean membrane conductance and mean ζ-potential across varying pH environments. The scatter plot shows the trajectory of intact (green) and permeabilized (orange) MCF-7 and MDA-MB-231 cells. Data points represent the mean values for specific pH conditions (labeled), and Spearman’s rank correlation coefficient (ρ) indicates the strength of the monotonic relationship.

Spearman correlation was performed to assess the relationship between *V_m_* and ζ-potential (Figure 6). A very weak negative correlation was observed for intact MDA-MB-231 cells (*r* = −0.14, *p* = 0.78), whereas a very weak positive correlation was observed in permeabilized cells (*r* = 0.071, *p* = 0.91); however, neither correlation was statistically significant. On the other hand, intact MCF-7 cells showed a statistically significant, strong negative linear relationship (*r* = −0.79, *p* = 0.048), whereas permeabilized MCF-7 cells exhibited a moderate, non-significant negative linear relationship (*r* = −0.54, *p* = 0.236). The relationship between *V_m_* and ζ-potential was generally negative across most pH values. However, at specific pH levels, this trend reverses, showing a positive correlation. These acidic pH points appear as outliers in the overall dataset, deviating from the main pattern observed at neutral and alkaline pH. Meanwhile, *G_eff_* and ζ-potential (Figure 7) only exhibited a significant monotonic association in intact MCF-7 cells.

## 4. Discussion

The ability of cells to adapt to variations in exogenous pH (pHe) is a crucial feature of their survival and progression in diverse environments. pHe was introduced as a controlled exogenous variable across a defined experimental range. This study investigated the regulation of membrane potential (*V_m_*) in breast cancer cells across a wide pH range (3.0–9.2), using both intact and permeabilized cells. Although the extracellular conditions may vary over time under standard incubator conditions, the cells were maintained under the specified conditions for 24 h, which encompasses at least one full cell cycle for both cell lines studied. This allows for meaningful assessment of pH-dependent responses within a biologically relevant timescale. DEP was used to estimate *V_m_* with a maximum difference of 6.3 mV from the mean published values [21]. Permeabilized cells were used as a control to eliminate the contribution of plasma membrane integrity and associated signaling. Therefore, electrophysiological changes observed in intact cancer cells but not in permeabilized cells indicate that the response to pHe is an active, regulated process involving membrane-bound ion channels, rather than a passive result of ionic shifts [32,33].

### 4.1. pH-Dependent Behavior Exhibits a Threshold at pH 6.7

Analysis of the data of the different parameters presented in the Results section shows an interesting trend. In almost all cases, the difference between cell response above pH 6.7 is consistent, with no significant differences; similarly, the measurements below this threshold generally exhibit no significant changes. However, significance does exist between the cells cultured at pH below this threshold from those cultured above it. This finding suggests an interpolated inflection point near pH 6.7; therefore, we classify this region as a transition zone, rather than a definitive, high-resolution biological threshold. The identification of the inflection points near pH 6.7 is robustly consistent with the established literature on the physical realities of solid tumor microenvironments. Mechanistically, this transition zone correlates closely with the activation kinetics of acid-sensing ion channels (ASICs) [35]. In this section, the data from these groupings are presented. The observed pH-dependent shifts in the two cell lines and the differences observed between intact and permeabilized cells suggest that the cells have adapted to growth in different conditions, particularly those grown in acidic conditions compared to neutral pH and above, which demonstrate similar responses, which in turn suggests that alkaline conditions do not change the cell from pH neutral behavior. The mechanism of adaptation may be via two routes; either the majority of the cells change behavior by altering their electrophysiological functionality (e.g., through the expression or activation of specific channels), or there may be an element of selection, where the altered environment causes a portion of cells to become stressed and die, leaving a response subset to proliferate. Whilst we did not identify the means by which the cells arrived at the state at time of analysis, the populations at point of analysis did exhibit common characteristics suggesting a population-level response.

By grouping the data from Figure 2, Figure 3, Figure 4 and Figure 5 into “acidic” and “neutral-basic” groups, we are better able to identify these trends. These replotted data can be seen in Figure 8, Figure 9, Figure 10 and Figure 11.

### 4.2. The Effect on Permeabilization on Cytoplasm Conductivity and Membrane Potential

**Figure 8:**
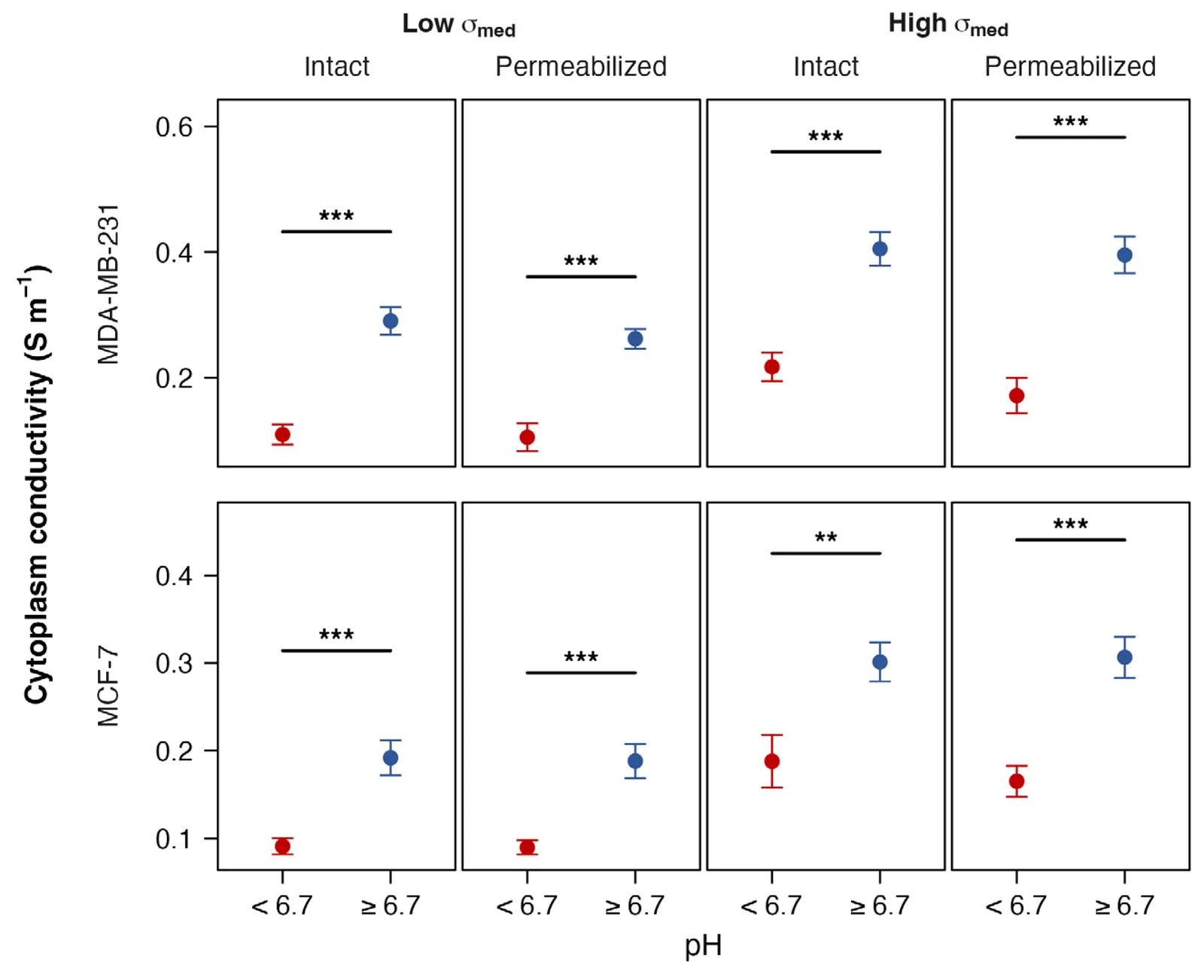
The cytoplasm conductivity of cells cultured in high pH (≥6.7, blue) and low pH (<6.7, red). Two-tailed *t*-tests assessed annotated *p*-values significance within each facet; annotated *p*-values indicate the magnitude of pH-induced differences (** *p* < 0.01; *** *p* < 0.001).

Both cell lines exhibited similar behavior in *σ_cyto_*, with values of cells from alkaline pH being typically 1.5× to 3× those grown in acidic pH as shown in Figure 8. In both intact and permeabilized MDA-MB-231, the values of *σ_cyto_* between permeabilized and non-permeabilized cells (as confirmed using Trypan blue) were similar, save for a reduction observed in permeabilized cells at pH in low-conductivity media, and low pH in high-conductivity media. This same trend was observed in MCF-7; the plots of the two cell lines were similar, except that permeabilized MCF-7 cells exhibited an unusual peak in conductivity at pH 7.4 in both conductivities.

This result is significant for two reasons. The first is that it demonstrates that pH plays an important role in setting *σ_cyto_*, which should be considered in DEP experiments in media of different pH. Secondly, it suggests that for most of the pH range, the calculated value of *σ_cyto_* does not depend on membrane permeabilization. The persistence of *σ_cyto_* after disruption of membrane integrity in permeabilized cells suggests that in these cells, the electrostatic behavior that is interpreted by the Clausius–Mossotti model as arising from intracellular conductivity is governed by factors other than free ions in the cytoplasm.

The similarities in *σ_cyto_* before and after permeabilization may reflect the way the membrane potential is generated, particularly at low conductivities. The derived spectra do not generally resemble either apoptotic cells (e.g., [36]) or heat-killed cells (e.g., [37]), suggesting the lack of difference is not due to non-permeabilized cells being killed by persistent exposure to low pH. Instead, the similarities may arise from functional changes in ion channel activity. Under our experimental conditions the extracellular concentrations of chloride and sodium are close to, or lower than, intracellular concentrations of the same ions; consequently, the membrane potential in these regimes may, for example, depend entirely on the functionality of potassium transport in these cells. Where non-permeabilized cells have high K^+^ conductivity at low ion concentrations, then the intracellular and extracellular ions may indeed be similar in both the permeabilized and non-permeabilized cases. Alternatively, it may suggest that intracellular conductivity is driven more by Donnan equilibrium processes than simple free cytoplasm ions, such that permeabilization does not alter the fixed internal charge (either in macromolecules such as proteins or on the inner membrane surface) that is responsible for either *σ_cyto_* or *V_m_*. While Donnan equilibrium has long been recognized as a fundamental determinant of intracellular ionic distribution, its contribution to dielectric measurements of *σ_cyto_* remained unreported. These findings suggest that *σ_cyto_* should not necessarily be interpreted solely as a measure of free, mobile intracellular ions, raising the possibility that the intracellular fixed-charge environment also contributes to the measured electrical phenotype. If validated, this interpretation would broaden the physiological significance of *σ_cyto_*, and such a perspective has important implications for impedance-based cell characterization, as changes in *σ_cyto_* could reflect alterations in intracellular macromolecular organization and ionic homeostasis. Trypan blue testing was used to confirm the integrity of the membrane in intact cells and membrane disruption in permeabilized cells. Similarly, there was an observed increase in *σ_cyto_* after permeabilization of MCF-7 cells observed between pH 6.7 and pH 7.4. This is surprising, but was observed across multiple biological repeats, suggesting it is not an artefactual phenomenon.

While previous studies have reported higher *σ_cyto_* in cancer cells compared to non-cancerous counterparts [33], the present study shows that for different cancer cell lines, pH response plays a greater role in modulating *σ_cyto_* than membrane integrity. These results suggest that elevated conductivity reported in cancer cells may partially reflect changes in membrane permeability, rather than exclusively intrinsic alterations in intracellular biochemistry. MDA-MB-231 (high metastatic potential) exhibited higher cytoplasmic conductivity compared to MCF-7 (low metastatic potential), which is consistent with a previous report in the literature [34].

**Figure 9:**
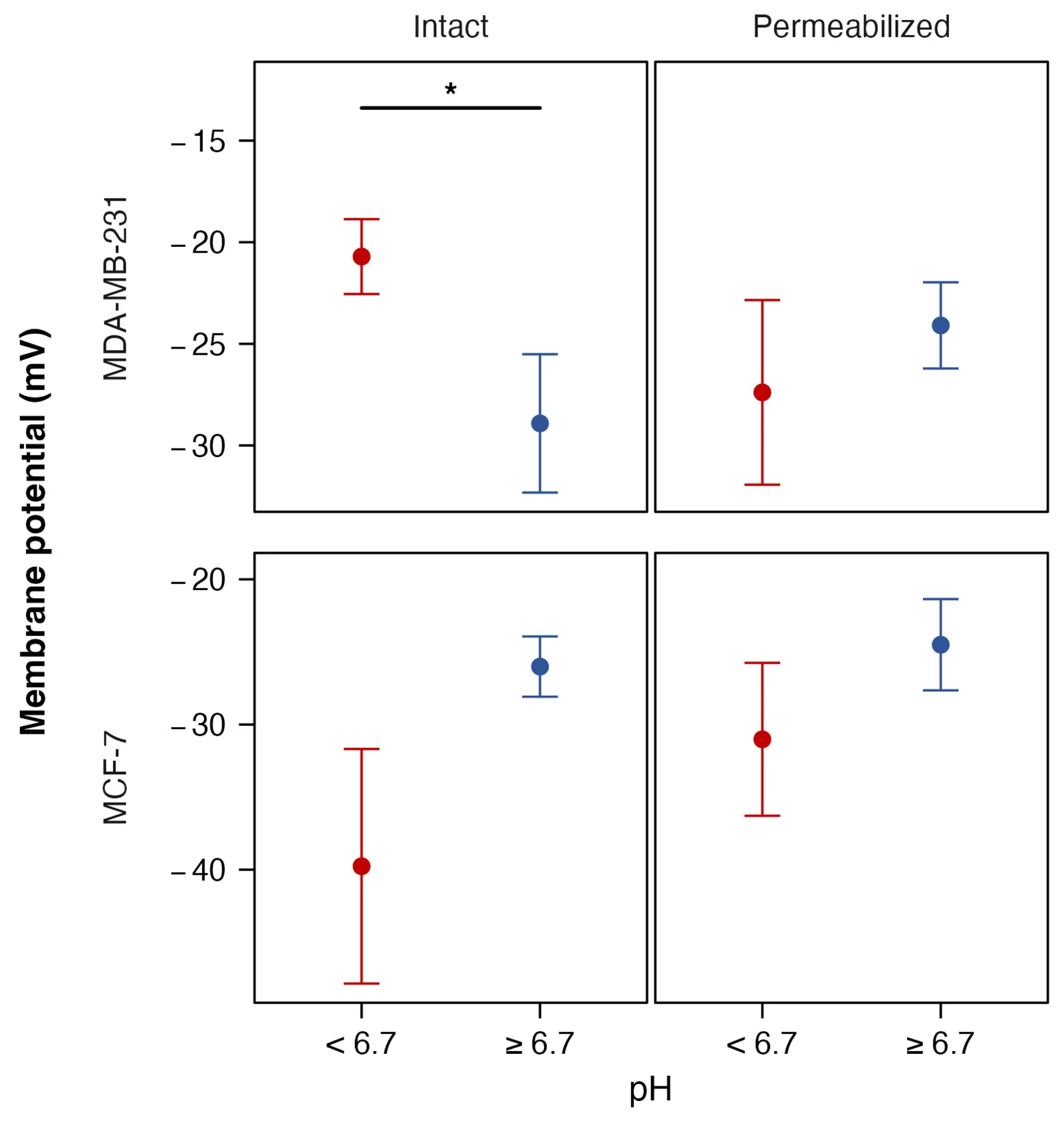
The estimated membrane potential of cells cultured in high pH (≥6.7, blue) and low pH (<6.7, red). Two-tailed *t*-tests assessed annotated *p*-values significance within each facet; annotated *p*-values indicate the magnitude of pH-induced differences (* *p* < 0.05).

We then used the two values of *σ_cyto_* taken at different values of *σ_med_* to produce a membrane potential value *V_m_* [21] shown in Figure 9. In intact MDA-MB-231 cells, the *V_m_* remained relatively stable across the pH range of 3.0–9.2, with no statistically significant changes between permeabilized and non-permeabilized cells. Considering the more general trend, the *V_m_* of the intact cells was depolarized by around 10 mV at pH 3.0–5.0 compared to higher pH, which was not observed in permeabilized cells, where MDA-MB-231 displayed substantial differences in membrane potential between the most acidic conditions (pH 3.0 and 4.0) and near-neutral pH (6.7). This suggests that in the absence of an intact membrane, the underlying intracellular ionic environment is more susceptible to extracellular pH changes. This further reinforces the suggestion that in acidic conditions both *σ_cyto_* and *V_m_* arise from fixed charge in the cell interior, such as the cytoskeleton, organelles, or even the inner surface of the membrane itself [38].

In contrast, intact MCF-7 exhibited a pronounced sensitivity to extracellular pH, with significant shifts in membrane potential observed at both acidic (pH 4.0 and 5.0) and alkaline (pH 8.4 and 9.2) ranges. This suggests that MCF-7 cells possess pH-responsive mechanisms, possibly involving ion channels or transporters, that actively modulate membrane potential in response to environmental pH. These differences were abolished following permeabilization, underscoring that the observed changes were dependent on active membrane function rather than passive ionic equilibration.

These contrasting patterns between subtypes highlight how different cancer cells may adopt electrochemical strategies to cope with acidic or alkaline microenvironments; notably, MDA-MB-231 cells have a higher metastatic potential than MCF-7 cells, which prefer to be located within a tumor microenvironment. It may even suggest that the change in cellular response to pH may be a trigger for encouraging cells to metastasize. Further investigation into the molecular basis of these subtype-specific responses, such as the role of ion channels, pH sensors, or membrane composition, may uncover therapeutic vulnerabilities linked to their electrophysiological behavior. Previous studies have reported membrane depolarization under acidic conditions [38], particularly in metastatic cancer cells.

### 4.3. The Effect of Permeabilization on ζ-Potential and G_eff_

**Figure 10:**
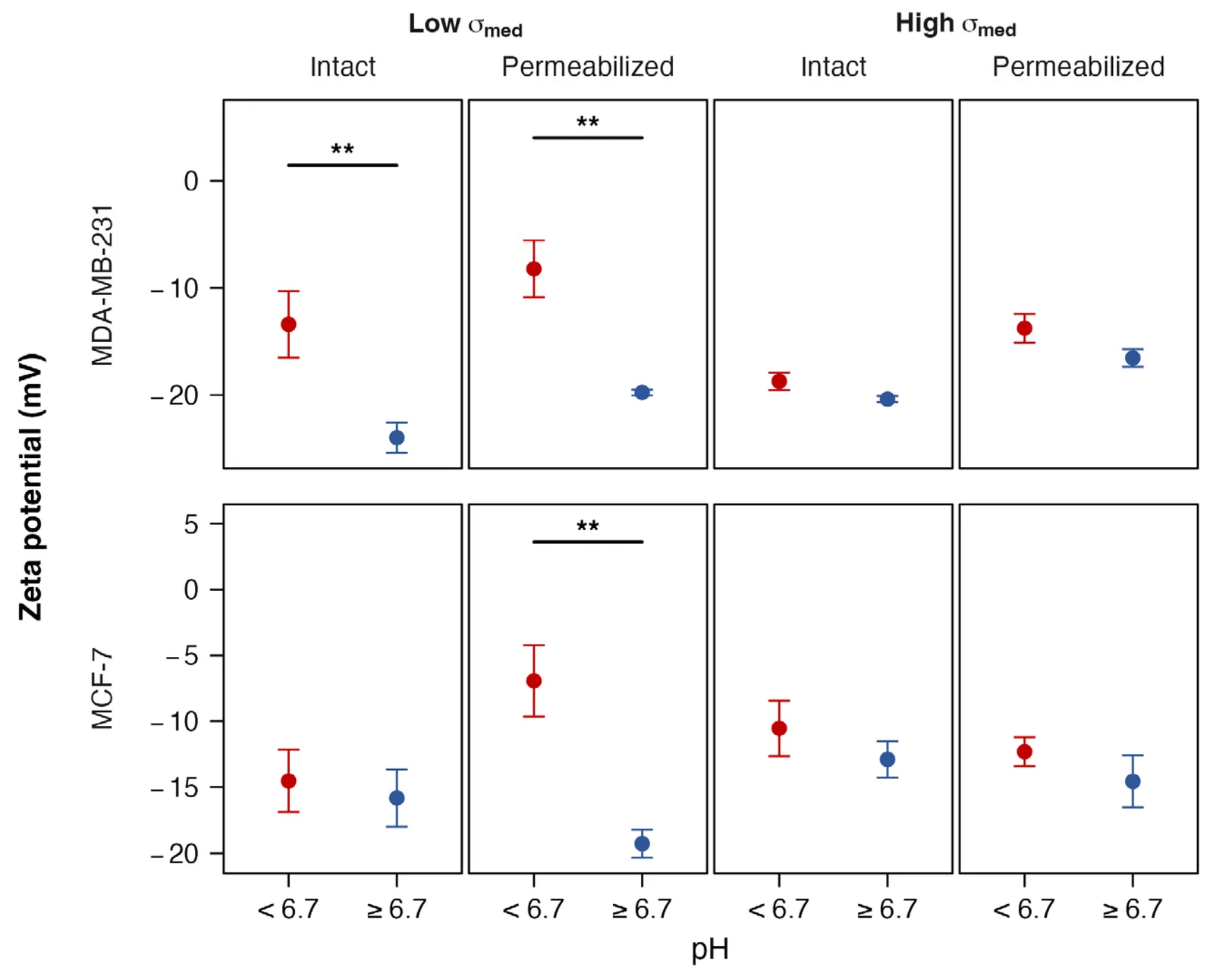
The ζ-potential of cells cultured in high pH (≥6.7, blue) and low pH (<6.7, red). Two-tailed *t*-tests assessed annotated *p*-values significance within each facet; annotated *p*-values indicate the magnitude of pH-induced differences (** *p* < 0.01).

We also examined two parameters associated with the cell surface, the ζ-potential and the effective membrane conductance per unit area, *G_eff_*. The ζ-potential behavior of both intact and permeabilized cancer cells was found to alter as a function of both medium conductivity and culture pH as shown in Figure 10. In low-conductivity medium, intact and permeabilized MDA-MB-231 followed the same trend of exhibiting a more negative ζ-potential as pH increased. Permeabilized cells consistently exhibited less negative ζ-potentials than intact MDA-MB-231 cells across all pH values. The standard model of the dependence of ζ-potential on pH is one characterized by constant values at the extremes of pH, with one or two transitions between these values at the isoelectric points of the groups on the surface, in line with the Henderson–Hasselbalch equation [39]. The ζ-potentials of both cell lines exhibit behavior that is generally in line with this prediction, particularly at lower conductivities where ζ-potential is in the −15 mV to −20 mV range at or greater than pH 4.0 but exhibits depolarization to around zero at pH 3.0, indicating the presence of an isoelectric point at this pH. This is in some ways surprising, since the presence of an isoelectric point would normally be identified when cells were measured at low pH, rather than cultured at low pH and measured under neutral conditions. The trend was not observed at higher medium ionic strength, with ζ-potential being hyperpolarized beyond −10 mV in MDA-MB-231 (both intact and permeabilized) and permeabilized MCF-7. Both intact lines in high-conductivity media exhibited a slight rise in ζ-potential at this pH, whereas the permeabilized did not. This is more likely to be due to the lower conductivity yielding an unexpected characteristic which is not present at higher salt concentration, though the level of concentration (and the difference in bulk ion concentration) is unlikely to lead to significant surface charge screening.

**Figure 11:**
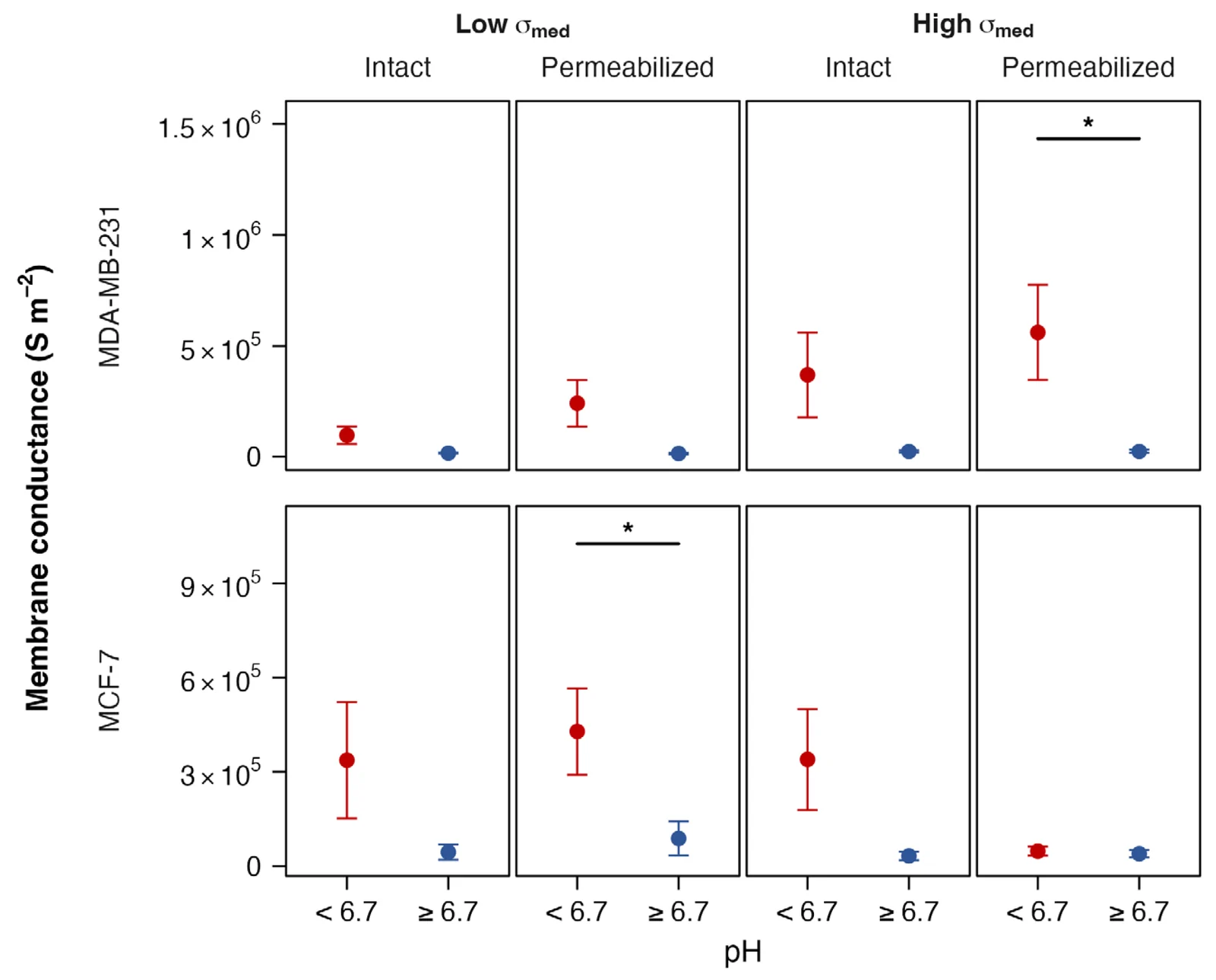
The membrane conductance (*G_eff_*) of cells cultured in high pH (≥6.7, blue) and low pH (<6.7, red). Two-tailed *t*-tests assessed annotated *p*-values significance within each facet; annotated *p*-values indicate the magnitude of pH-induced differences (* *p* < 0.05).

Whilst the ζ-potential depolarizes in cells from lower pH but remains more polarized at higher values, the converse was observed for *G_eff_* (Figure 11). Here, we find for all conditions an approximately exponential increase in *G_eff_* as pH is reduced below neutral, with *G_eff_* being at or near zero at higher pH. The two cell lines exhibited different relationships between intact and permeabilized states; for MDA-MB-231 cells, the permeabilized cells were more conductive than the intact cells, whereas the reverse was true for MCF-7. This result is in line with observations of a negative correlation relationship between *G_eff_* and ζ-potential observed in platelets [15], with *G_eff_* increasing as ζ-potential becomes less negative. When we analyzed the two parameters against each other in both cancer cells (Figure 7), a monotonic relationship was observed in intact MDA-MB-231 cells at high conductivity (with a Spearman correlation coefficient of 0.79 indicating that as *G_eff_* increases, ζ-potential becomes less negative); in all other cases, no correlation was observed because under most conditions, *G_eff_* was too low (being at or near zero) to form a linear relationship with a non-zero gradient.

One factor that should also be considered is that beyond permeabilization, Tween-20 may also alter the charge distribution on the membrane surface, such as by masking surface charges, altering the membrane or glycocalyx microstructure, or shifting the electrokinetic shear plane, all of which would alter ζ-potential. It is also worth considering that the observed effects do not derive from changes due to cell trypsinization, which has previously been shown not to affect cell electrical properties [40].

### 4.4. On the Relationship Between V_m_ and ζ-Potential

At both medium conductivities, the ζ-potential of MDA-MB-231 cells was on average less negative in intact cells than permeabilized cells (+4.4 mV at low conductivity, +4.2 mV at high conductivity), suggesting that membrane permeabilization does impact ζ-potential and that the cytoplasm content does impact the way in which cells respond to their electrical environment. It has been demonstrated in multiple cells [9,11,13,15,41] that a portion of *V_m_* appears at the cell surface in addition to the ζ-potential arising from cell surface charge. To test this, we examined the difference between *V_m_* and ζ-potential as a proportion of measured *V_m_* (assuming *V_m_* is the same at both conductivities), a parameter referred to as Ξ by Hughes et al. [13]. We found this value was consistent across both permeabilized cell types and both conductivities, averaging 0.36 ± 0.07, and showing a high degree of consistency with the 0.37 observed in red blood cells [13]. When we performed the same analysis for intact cells, we found substantial differences. The average value of Ξ in intact MDA-MB-231 was lower (0.21 in low conductivity, 0.18 in high conductivity). MCF-7 at low conductivity showed the common value of 0.37 at low conductivity, and a much higher dependence on Vm of 0.56 at higher conductivity. The value of Ξ was elevated across pH, with over 50% of measurements above 0.6 and the remainder around the standard value of 0.37. MCF-7 cells showed greater differences between intact and permeabilized cells, particularly at more acidic pH in lower-conductivity media where intact cell ζ-potential became increasingly polarized while permeabilized cells became depolarized, resulting in a ~25 mV difference at pH 3.

### 4.5. General Observations on Cell Behavior at High and Low pHe

When we examined the behavior of cells cultured across the pH spectrum, we noted distinct differences between values obtained from cells cultured at pH 6.7 and above and those at pH 5.0 and below. For example, the *V_m_* of cells at higher pH was around 50% higher than that at low pH in MDA-MB-231 cells; *σ_cyto_* was around 50–100% lower at low pH in MCF-7 across the two conductivities and around 100–200% lower in MDA-MB-231 under the same conditions; whilst *G_eff_* had near-zero values at neutral and alkaline pH, it was substantially higher at pH 3.0–5.0. When we grouped the low-pH and medium-high-pH values and tested for significance (two-tailed Student’s *t*-test following confirmation of normality by Shapiro–Wilk test), we found a high degree of significance between the two groups for both intact and permeabilized MDA-MB-231 for *σ_cyto_* (*p* < 0.0001) at both conductivities, ζ-potential (normal, *p* = 0.010; permeabilized 0.0023) at low conductivity, whilst significance was not observed at higher conductivity (*p* = 0.085, *p* = 0.0998 respectively). Similarly, membrane conductance was only significant for permeabilized cells at high conductivity (*p* = 0.037) but was near significance for low conductivity intact (*p* = 0.073) and permeabilized cells (*p* = 0.062). MCF-7 was also significant for all cases for *σ_cyto_* (*p* varying between 0.0001 and 0.0079), and permeabilized at low conductivity for *G_eff_* (*p* = 0.043) and ζ-potential (*p* = 0.0015). This suggests that MDA-MB-231 in particular exhibits a “low pH phenotype” that is significantly distinct from its neutral/alkaline type; MCF-7 exhibited the same effect in *σ_cyto_*, but this appears to have less impact on the surface characteristics, or the degree of variability observed in the MCF-7 data set makes identifying such a phenotype harder.

**Figure 12:**
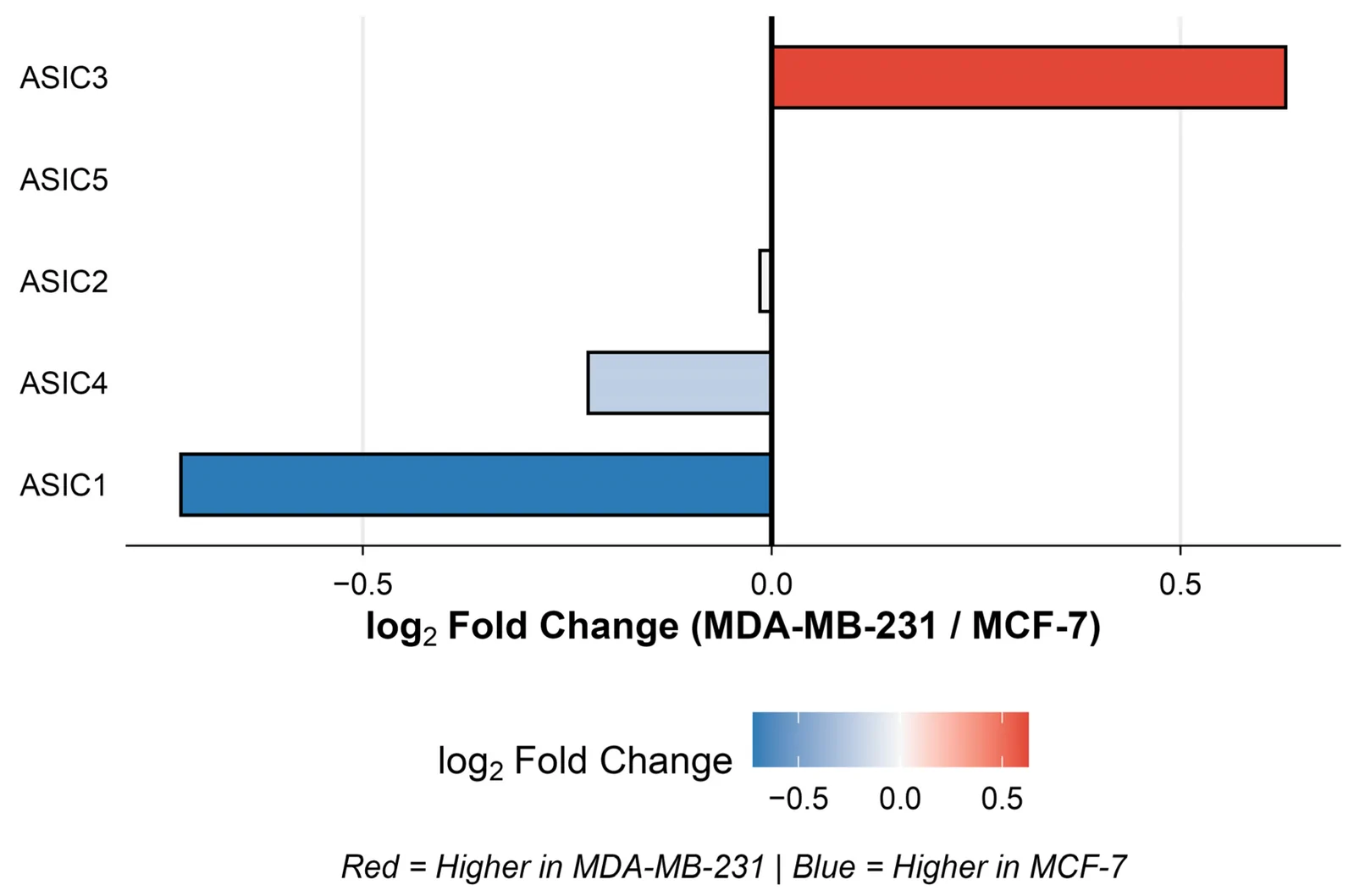
Subtype-dependent variation in acid-sensing ion channel expression. Bar plot illustrating log_2_ fold changes (MDA-MB-231/MCF-7) in both cancer cells. Each bar represents one gene, annotated by its channel family. Positive values (red) indicate upregulation in MDA-MB-231 cells, while negative values (blue) indicate higher expression in MCF-7 cells.

To explore further, we used publicly available baseline gene expression data for acid-sensing ion channel (ASIC) subtypes in both cell lines, as shown in Figure 12. These showed elevated acid-sensing ion channel (ASIC) expression, suggesting increased capacity for proton-gated cation conductance across the plasma membrane. In MCF-7, higher expression of ASIC1, ASIC2, and ASIC4 produces transient, threshold-activated currents that could briefly depolarize the membrane during acute acid stress, allowing *V_m_* to fluctuate with pHe [42]. Meanwhile, in MDA-MB-231 cells, ASIC3 dominance mediates sustained proton-gated conductance near physiological pH [43], maintaining a quasi-steady *V_m_* even as pHe varies, thereby buffering electrical changes via a continuous leak-like current. Following permeabilization, MCF-7 loses its exchanger- and ASIC-mediated regulation, collapsing to a passively driven *V_m_*. In contrast, the disruption of the MDA-MB-231 membrane may have led to ASIC3 no longer being electrically compensated by intact conductances, resulting in passive proton sensitivity and a pH-dependent *V_m_* shift.

The mechanisms by which *G_eff_* may alter are manifold. For example, alteration of surface charge near the isoelectric point would be expected to reduce surface charge, but we see elevated membrane conductance, implying elevated surface charge [23]. Alternatively, alteration of pH may alter membrane fluidity. As before, the interesting aspect of this is that measurements were all made at neutral pH, suggesting that cell alterations made under pH stress are retained following resuspension, or even that compensatory alterations are made to the cell membrane to maintain normality in low pH, leading to overcompensation where normality is resumed. The phenomenon of cytoplasm conductivity increasing with pH but membrane conductance decreasing suggests an inverse relationship between these two parameters, which has been reported in other cells, but this coupled with a decrease in ζ-potential is intriguing.

### 4.6. Integrative Model of Subtype-Specific Biophysical Dynamics Across a pH Spectrum

Under physiological baseline and mildly alkaline conditions, the two subtypes exhibit fundamentally distinct electrophysiological phenotypes. MDA-MB-231 exhibited higher cytoplasmic conductivity, a more hyperpolarized *V_m_*, and a significantly more negative ζ-potential compared to MCF-7, suggesting that even in the absence of environmental stress, MDA-MB-231 maintains a distinctly higher baseline of internal ionic mobility and a more densely charged surface interface. As the cellular environment shifted to high acidity, the management of extracellular protons (H^+^) suggest different adaptive mechanisms in MDA-MB-231 to MCF-7. While acidity broadly results in surface protonation, MDA-MB-231 exhibited *V_m_* depolarization and a significant decrease in the negativity of ζ-potentials. We propose that a coordinated membrane-to-cytoplasm cascade governs MDA-MB-231. The extreme protonation at the surface acts as a direct ligand for acid-sensing channels. The gating of these channels allows for an influx of cations, fundamentally collapsing the resting electrochemical gradient to drive the unique *V_m_* depolarization observed in MDA-MB-231. This breakdown of membrane insulation concurrently elevates *G_eff_* and forces a recalibration of internal ionic strength, reflected in MDA-MB-231′s characteristically high cytoplasmic conductivity; since the mechanism involves alteration of *V_m_*, the concomitant change in ζ-potential alters the extracellular ion concentration; specifically, depolarization from negative ζ-potential would reduce surface concentrations of cations at the surface, including H^+^. The persistence of this effect after permeabilization suggests that changes brought about by culture at low pH are structural, rather than simply being due to altered cytoplasm ion content.

### 4.7. Limitations and Future Directions

While the present study establishes a comprehensive electrical characterization of breast cancer cell lines cultured across varying extracellular pH conditions, several conditions and limitations should be considered when interpreting the findings. The pH range investigated extended to highly acidic conditions (pH 3.0–4.0), which lie beyond the typically observed acidity in the tumor microenvironment. These conditions were included to define the limits of the cells’ electrical responses, rather than to replicate physiological conditions. Likewise, electrical characterization required a low-conductivity suspension medium, a standard requirement for dielectric measurement that minimizes background ionic contributions, although it does not reflect the conductivity of biological fluids. Moreover, the study did not directly quantify intracellular ion distributions or fixed charge densities; the findings of the role of Donnan equilibrium in *σ_cyto_* should be regarded as a plausible mechanistic framework, rather than establishing a definitive role for Donnan equilibrium. Finally, it is important to consider these findings in light of the fact that whilst cells were *cultured* across a range of values of pH, they were all *measured* at neutral pH.

## 5. Conclusions

The main objective of this study was to characterize the electrophysiological response of breast cancer subtypes. This work demonstrates the distinct bioelectric signature of the highly aggressive MDA-MB-231 and the less aggressive MCF-7 cells as a function of pH. The findings presented here highlight the ability of cancer cells to modulate their bioelectric properties under varying pH conditions. Overall, MDA-MB-231 exhibited higher cytoplasmic conductivity, accompanied by a more negative membrane and ζ-potential compared to MCF-7. This sheds light on specific electrical properties that could aid in the survival and metastasis of highly aggressive cells. Acidic conditions were also associated with decreased negativity of the ζ-potential and *V_m_* depolarization, a finding that is unique to intact MDA-MB-231. Collectively, these observations demonstrate the subtype-specific adaptation of breast cancer cells to acidic microenvironments. The identification of the unique electrical response of cells to pHe can be leveraged to open a new therapeutic avenue that targets aggressive cancer subtypes. Moving forward, integrating bioelectric profiling with genomic analysis could provide a comprehensive understanding of tumor aggressiveness.

## Data Availability

Data are available on request from Michael Pycraft Hughes, michael.hughes@ku.ac.ae.
